# Tropomyosin 1: Multiple roles in the developing heart and in the formation of congenital heart defects

**DOI:** 10.1016/j.yjmcc.2017.03.006

**Published:** 2017-05

**Authors:** Jennifer England, Javier Granados-Riveron, Luis Polo-Parada, Diji Kuriakose, Christopher Moore, J. David Brook, Catrin S. Rutland, Kerry Setchfield, Christopher Gell, Tushar K. Ghosh, Frances Bu'Lock, Christopher Thornborough, Elisabeth Ehler, Siobhan Loughna

**Affiliations:** a School of Life Sciences, University of Nottingham, UK; b Laboratory of Genomics, Genetics and Bioinformatics, Hospital Infantil de México Federico Gómez, Mexico; c Department of Medical Pharmacology and Physiology, School of Medicine, University of Missouri, USA; d School of Veterinary Medicine and Science, University of Nottingham, UK; e School of Life Sciences Imaging, University of Nottingham, UK; f East Midlands Congenital Heart Centre, Glenfield Hospital, Leicester, UK; g Randall Division of Cell and Molecular Biophysics, The Cardiovascular Division, King's College London, UK

**Keywords:** Cardiac development, Congenital heart defects, Structural protein, Tropomyosin 1

## Abstract

Tropomyosin 1 (TPM1) is an essential sarcomeric component, stabilising the thin filament and facilitating actin's interaction with myosin. A number of sarcomeric proteins, such as alpha myosin heavy chain, play crucial roles in cardiac development. Mutations in these genes have been linked to congenital heart defects (CHDs), occurring in approximately 1 in 145 live births. To date, *TPM1* has not been associated with isolated CHDs. Analysis of 380 CHD cases revealed three novel mutations in the *TPM1* gene; IVS1 + 2T > C, I130V, S229F and a polyadenylation signal site variant GATAAA/AATAAA. Analysis of IVS1 + 2T > C revealed aberrant pre-mRNA splicing. In addition, abnormal structural properties were found in hearts transfected with TPM1 carrying I130V and S229F mutations. Phenotypic analysis of TPM1 morpholino-treated embryos revealed roles for *TPM1* in cardiac looping, atrial septation and ventricular trabeculae formation and increased apoptosis was seen within the heart. In addition, sarcomere assembly was affected and altered action potentials were exhibited. This study demonstrated that sarcomeric *TPM1* plays vital roles in cardiogenesis and is a suitable candidate gene for screening individuals with isolated CHDs.

## Highlights

• Four mutations identified in the *TPM1* gene; IVS1 + 2T > C, I130V, S229F and GATAAA/AATAAA.
• In vitro analysis of IVS1 + 2T > C revealed aberrant pre-mRNA splicing.
• I130V and S229F mutations caused abnormal structural properties in the sarcomere.
• Reduced TPM1 expression during early cardiogenesis causes aberrant gross morphology.
• Apoptosis, sarcomere assembly and cardiac conduction were also affected.

## 1 Introduction

The heart is first seen as a tube early in development, which undergoes a series of complex processes to form a four-chambered structure. Looping of the chicken primitive heart tube is initiated from Hamburger and Hamilton (HH) 10 (human gestation day 22) [1], [2]. This tube subsequently divides into chambers by septation, initiated in the primitive single atrium from the dorso-cranial wall around HH14 (4th week in human) followed by septation of the common ventricle [3]. In humans, congenital heart defects (CHDs) occur in approximately 1 in 145 live births [4]. Although most cases of CHDs are thought to have complex etiology, some cases segregate through families following Mendelian patterns of inheritance. Studying the genetic causes of the Mendelian variety could potentially unravel the biology of this disease, which, in turn, could be applied to the prevention and treatment of complex-etiology CHD. The molecular genetics of Mendelian CHDs are being elucidated, with mutations found in several genes, including transcription factors NKX2 homeobox 5 (*NKX2.5*) and T-box 5 (*TBX5*), and structural proteins myosin heavy chain 6 (MYH6), MYH7 and alpha cardiac actin [5], [6], [7].

The cardiac conduction system (CCS) also appears early in cardiogenesis with the development of a primordial pacemaker, and subsequent formation continues with differentiation of the Purkinje fibres still ongoing around birth. The CCS is also susceptible to defects with associated mutations in several genes; malformations may occur in isolation or in association with structural defects such as atrial septal defects (ASDs) [8]. Although much progress has been made in the understanding of cardiogenesis and its contribution to CHDs and CCS malformations, the causative genetic determinants remain unknown in most cases.

Cardiomyopathies are contractile diseases of the heart, which are associated with heart enlargement and dysfunction. The most common types are hypertrophic and dilated cardiomyopathy (HCM and DCM, respectively). HCM is defined as a notable thickening of the ventricular wall and DCM by ventricular dilatation and decreased contractile function [9]. Mutations in a range of sarcomeric genes have been associated with both HCM and DCM, including *Tropomyosin 1* (*TPM1*) [10], [11]. Further, a number of genes encoding structural proteins have been associated with both cardiomyopathies and CHDs in the same individuals, such as *TPM1*, *MYH7* and *alpha cardiac actin* [6], [12], [13], [14]. Fewer sarcomeric genes, such as *MYH6* and *alpha cardiac actin*, have been associated with isolated CHDs [5], [7], [15], or with the developing heart [16].

Tropomyosin regulates contraction of the sarcomere, through direct interaction with actin and troponin T [17]. In the presence of elevated levels of intracellular calcium (Ca^2 +^), Ca^2 +^ binds to troponin C of the troponin complex inducing conformational changes that moves tropomyosin across the actin filament, exposing the myosin-binding sites, allowing the globular myosin heads to interact. This produces movement of the thick and thin filaments relative to each other, resulting in sarcomere shortening and hence muscle contraction [18], [19]. Despite this essential interaction, roles for TPM1 in the developing heart are poorly understood, with isolated CHD causative mutations previously not described within the literature. Because of the aforementioned involvement of other genes encoding components of the thick and thin filaments of the cardiac sarcomere in both cardiomyopathy and heart development, we aimed to screen *TPM1* in a cohort of patients with diagnosed CHDs (presented in this report). Our initial results prompted us to investigate developmental roles of TPM1 and its associated thin filament protein cardiac Troponin T (cTNT) [16].

Here we report the screening of the *TPM1* gene in 380 patients with various CHDs. Four variants were detected. A splice donor site mutation resulted in abnormal splicing of pre-mRNA, while two non-synonymous mutations failed to incorporate with the sarcomeric protein cardiac troponin T in the sarcomere. Additionally, we detected a polyadenylation site variant. We showed that upon treatment with TPM1-specific morpholino oligonucleotides, the atrial septum and ventricular trabeculae developed abnormally. Additionally, some hearts showed abnormal looping. Although mature sarcomeres formed normally, there were fewer mature structures and increased apoptosis in the TPM1 morpholino-treated hearts. Further, the action potential (AP) of the cardiomyocytes was affected. These data suggest that TPM1 is essential for normal heart development and contractile function. Screening detected four variants in the *TPM1* gene, of which three we predict would lead to a pathological phenotype; we therefore consider *TPM1* a gene worthy of screening patients afflicted with CHDs or conduction anomalies.

## 2 Materials and methods

### 2.1 Phenotyping of patients

Patients with any form of CHD were recruited. All volunteered to participate and were under the routine care of the East Midlands Congenital Heart Centre, Leicester, UK. All patients had their CHD diagnosed fully by echocardiography and any other diagnostic modalities needed. All phenotyping, non-cardiac diagnoses and family histories were confirmed by one of the authors (Bu'Lock) who is an experienced Consultant Congenital and Paediatric Cardiologist.

### 2.2 Mutational dHPLC analysis

dHPLC analysis was completed as previously described [20] under consent from participants and according to local ethics committees. Samples of peripheral blood were collected from all participants. Genomic DNA was purified from the blood samples using QIAmp DNA blood Midi Kit (Qiagen). Control DNA from anonymous human UK blood donors was obtained from the European Collection of Cell Culture (Salisbury). The mutational analysis by dHPLC was performed by designing 14 PCR assays to cover the exons of the *TPM1* gene and their surrounding sequences (see Table S1). The melting temperature (Tm) for each amplicon was calculated with the following formula: Tm = 63.728 + (0.41 × %GC) – (600/length) where %GC = percentage GC of the primer; length = length of the primer in nucleotides. The annealing temperature used was the average of the two primer Tms + 3 °C.

PCR products were obtained by standard protocols from patient genomic DNA. After amplification, a final hybridization step was carried out, starting at 95 °C and reducing the temperature by 1.5 °C per minute to 25 °C in order to favour the formation of heteroduplexes. The sequence of each amplicon was analysed using the Navigator software (Transgenomic) to determine the melting profile of each DNA fragment and select the optimal temperatures for dHPLC. PCR products were processed using the dHPLC WAVE System (Transgenomic). PCR products from samples showing a dHPLC trace suggestive of variation were amplified again and sequenced using standard protocols. Potentially deleterious variants were screened by dHPLC in 380 individuals with a CHD and 384 ethnically matched control subjects.

### 2.3 Homology molecular model of the S229F TPM1 protein

The molecular model of the S229F TPM1 mutant protein was made by comparing human wild-type TPM1 protein with the known structure of other tropomyosin proteins [21] (Protein Data Bank ID:1C1G). Images were prepared using PyMOL Molecular Graphics System software v0.99 (DeLano Scientific).

### 2.4 Splicing-donor site mutation of TPM1

Genomic DNA was obtained from patient A with a heterozygous splicing-donor site mutation at exon1-intron1 (described in Section 2.2). DNA was also obtained from a control subject. PCR products were obtained and subcloned into pcDNA3.1(+) (Invitrogen). COS7 cells were transfected with each construct using Polyfect Transfection reagent (Qiagen) and incubated for 48 h. Following RNA extraction, reverse transcription was performed using 0.5 μg of RNA per reaction. A primer pair specific to *TPM1* 5′ upstream and exon2a were used to detect *TPM1* products. Full procedure is available in Supplementary material and methods.

### 2.5 Missense mutations of TPM1α

Full length human *TPM1α* cDNA was amplified using specific primers. The *TPM1α* product was cloned using the StrataClone PCR cloning set (Agilent Technologies) and subcloned into pEGFP-C1 vector (Clontech). The I130V and S229F missense mutations were introduced to the *TPM1α* sequence using site-directed mutagenesis using primers in Table S2. See Supplementary materials and methods for further information.

### 2.6 Chick embryo care and maintenance

White fertile chick eggs (*Gallus gallus*, Dekalb White strain; Henry Stewart) were incubated in a humidified chamber at 38 °C under constant rotation [1]. Before treatment, the embryo was exposed *in ovo*, and 3–5 ml of albumin was removed with a syringe to separate the embryo from the overlying allantoic and vitelline membranes, and both membranes were carefully removed. After treatment, eggs were resealed with masking tape and reincubated until harvesting. Studies were performed within national (UK Home Office) and institutional ethical regulations.

### 2.7 Transfection of embryonic chick hearts with TPM1 missense mutant constructs

Fertile chick eggs were incubated, as described in Section 2.6, until HH10/11 [1], approximately 48 h. Following a published protocol [22], a transfection mixture was prepared as follows: 4 μg of either GFP/TPM1-WT, GFP/TPM1-I130V or GFP/TPM1-S229F construct, 10 μl Lipofectamine in 50 μl OptiMEM, (Life Technologies), and 0.5 μl Fast Green for visualisation. 4 μl of the transfection mixture was loaded into a glass needle and attached to a picospritzer (Parker Hannifin). Fifteen injections of 3 nl transfection reagent was injected into the embryonic pericardial space. Following incubation for a further 48 h, embryos were extracted, placed in 4 °C PBS and checked for GFP fluorescence (SV11 stereomicroscope, Zeiss). Embryos were fixed in 4% paraformaldehyde (PFA) for 30 min, washed with PBS and transferred to 30% sucrose. Embryos were mounted in OCT, orientated right side facing down and stored at − 80 °C until cryosectioned at 10 μm (Leica) and stored at − 20 °C.

### 2.8 Immunofluorescence and imaging of hearts transfected with TPM1 mutant constructs

Cryosections were processed using standard immunohistochemical procedures (Supplementary material and methods). Images were collected using a DeltaVision Elite microscope (GE Healthcare Life Sciences) as *Z*-stacks in two separate colour channels to monitor emission from troponin and GFP (detailed methods and parameters in Supplementary materials and methods). Subsequent analysis of images was performed in Fiji [23]. Representative fields of view (Fig. 3B) were selected by choosing single *Z*-planes that showed clear periodic troponin sarcomeric marker.

### 2.9 Morpholino design

Three morpholinos were designed against *TPM1* [24]: 5′TCCCGCGAGAAGTACAGCCGAAATC3′ to translational start-site (ATG); 5′GCTCTCAGTGCAGACCTGCTTGCTC3′ designed to the exon1-intron1 (E1I1) splice-site; 5′TTCCCTGTGTCCCAAAACTGACCTC3′ to the exon4-intron4 (E4I4) splice-site (GeneTools). A standard control (SC) morpholino was used as a positive control (5′CCTCTTACCTCAGTTACAATTTATA3′). Morpholinos were fluorescein tagged and underwent strict sequence similarity testing ensuring gene specificity.

### 2.10 Application of TPM1 morpholino and embryo isolation

Morpholino application was performed as previously described [25] using fertile chicken eggs at HH10/11 [1]. Embryos were harvested at HH19 (81 h) using a SV11 stereomicroscope (Zeiss) to determine morpholino uptake, stage embryos, and perform external phenotypic analysis [26]. Data recording the numbers of chick embryos alive at harvesting and those that were ‘morpholino positive’ were collected and statistically analysed (see below); n = 74 untreated (UT), 56 SC and 96 TPM1-morpholino (TPM1-MO) embryos. Embryos isolated for internal phenotypic analysis were fixed in 4% PFA as previously described [27]. See Supplementary material and methods for further information.

### 2.11 Systematic random sampling

Systematic random sampling [28] was used to quantify cardiac tissue proportions (HH11/19). Three groups were analysed; UT (n = 6), SC (n = 5) and TPM1-MO (n = 13) and underwent statistical analysis (see below). A 96 point grid was placed over every third section throughout the heart, and the tissue type on each point was identified (12,225 points counted).

### 2.12 Immunofluorescence and electron microscopy

Hearts processed for immunofluorescence (n = 8 TPM1-MO, n = 3 control) and transmission electron microscopy (n = 9 TPM1-MO, n = 8 control) were performed as previously described [27]; see also Supplementary material and methods.

### 2.13 Proliferation and apoptosis

TPM1-MO (n = 7), SC (n = 3) and UT (n = 3) embryos were fixed in 10% neutral buffered formalin and embedded in paraffin wax. Embryos were sectioned at 5 μm using DSC1 microtome (Leica) and every 8th section was collected on poly-l-lysine slides (Sigma). Sections were de-paraffinised in xylene and rehydrated through graded alcohols. Proliferation was detected using Zymed's Proliferating Cell Nuclear Antigen (PCNA) staining kit according to manufacturer's instructions (Zymed Laboratories). For apoptosis detection, ApopTag® Peroxidase In Situ Apoptosis Detection Kit (Millipore) was used according to manufacturer's instructions, mounted in VECTASHIELD HardSet Mounting Media with DAPI (Vector laboratories) and imaged using the Axioskop 2 (Zeiss) and Openlab (PerkinElmer). Total cell number (DAPI plus DAB reactive cells) was used in order to calculate the ratio and proportions of proliferating/apoptotic cells for each heart.

### 2.14 Isolation of cell micromass from embryonic chick hearts, morpholino treatment and analysis

Cardiac cells were isolated from day 5 embryonic chicks using previously described methods [29]. After 24 h, 10 mM of TPM1-MO was added to the cardiac cell micromass and cells were incubated for 48 h. Cells were fixed in 4% PFA and immunostained with anti-troponin T antibody (DSHB). Cells were visualized using the DMIRE2 inverted microscope (Leica). Cardiomyocytes treated with TPM1-MO were classified into four stages depending on the degree of sarcomeric maturity (Fig. 7A). Stage one consisted of cells with positive staining around the periphery of the cell but no sarcomeric structures can be clearly seen, especially around the nucleus (Fig. 7Aa). Stage two cardiomyocytes contain assembled sarcomeres; however, they appear thin and disorganised (Fig. 7Ac). Stage three cardiomyocytes present with organised sarcomeres that appear thin but are organised and run parallel to one-another (Fig. 7Ae). Finally, stage four cardiomyocytes contain mature sarcomeres with thick bands running through the cell (Fig. 7Ag). See Supplementary material and methods for detailed protocol.

### 2.15 Electrical activity

Intracellular recordings from TPM1-MO embryos (HH11/19; n = 3) and control (n = 3) spontaneous beating hearts were performed as previously described [27], [30]. See Supplementary material and methods for details.

### 2.16 Statistics

Levene's test for equality of variances was used followed by a *t*-test for equality of means using SPSS V21.0 (SPSS); *P* < 0.05 was considered as significant. Where appropriate, the mean ± SEM was calculated.

## 3 Results

### 3.1 Mutations in TPM1 are associated with CHDs

**Fig. 1:**
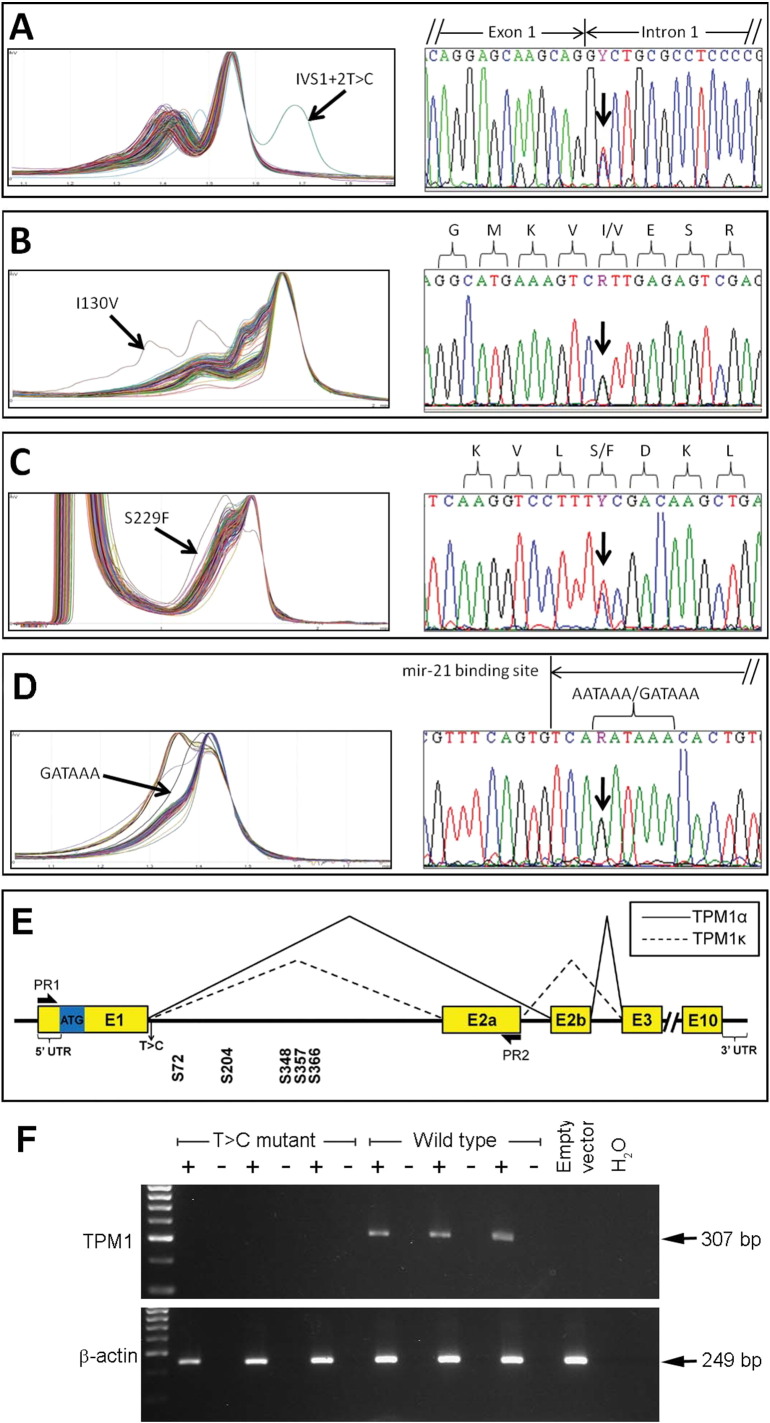
Sequence traces demonstrating *TPM1* mutations in patients with CHDs and the effect of a splice-donor site mutation on splicing. A, B, C, D. dHPLC traces and electropherograms corresponding to the CHD samples with a *TPM1* gene mutation. The right panel shows the nucleotide substitution for each variant. Arrows demarcate variation in the DNA sequence observed. E. Schematic illustration of *TPM1*. Two variants were found in intron1-2a: IVS1 + 2T > C (indicated as T > C). Primers were designed to obtain amplicons containing these variants from the genomic DNA of patient A (PR1 in the 5′ upstream region and PR2 in exon 2a). Five stop codons are present within intron 1-2a (S72, S204, S348, S357 and S366) in the 1st open reading frame. ATG, ATG start-site; E, exon; UTR, untranslated region. F. PCR showing the splicing of *TPM1* containing IVS1 + 2T > C mutation versus the wild-type. The wild-type mRNA splices normally, giving an expected band size of 307 bp (+ lane), the IVS1 + 2T > C mutant produce no splice product. β-actin acted as a loading control (249 bp). The –ve RT (− lane) and PCR controls (H_2_0) showed no signal.

To determine whether CHD patients carry *TPM1* mutations, 380 patients with a range of heart phenotypes were screened. Four variants were identified: one splice-donor site mutation, a polyadenylation signal variant and two non-synonymous missense mutations. The splice-site mutation was identified in patient A with tetralogy of Fallot (TOF; characterized by outlet ventricular septal defect with anterocephalad deviation of the outlet septum resulting in the aortic valve over-riding the interventricular septum, pulmonary stenosis and right ventricular hypertrophy). This mutation occurred in the donor-site of intron1/2, and consisted of a thymine to cytosine transition in the second nucleotide of the consensus (IVS1 + 2T > C; Fig. 1A). Patient B had pulmonary atresia (malformed pulmonary valve) and an intact interventricular septum with a hypoplastic right ventricle (and of necessity an atrial septal defect and persistent *ductus arteriosus* [elements of the fetal circulation required for postnatal survival to permit treatment; a communication remaining between the aortic arch and pulmonary trunk]). At the time of diagnosis, an A > G transition was detected (Fig. 1B). The mutant allele encodes a TPM1 protein where an isoleucine is replaced by a valine residue at position 130 (I130V). A C > T transition, encoding a mutant S229F protein, was found in patient C with an ASD (Fig. 1C). These three mutations mentioned above are not present on any public databases. For all three cases we were not able to assess the de novo status of the variants as we do not have access to one or both parental samples.

The polyadenylation signal variant replaces adenine for guanine in the first position of the consensus (GATAAA/AATAAA). It was discovered in patient D with two ASDs and secondary dilation of the right heart chambers (Fig. 1D). It is likely that the right heart dilatation is secondary to the left to right atrial shunt, although a right heart myocardial abnormality in association with atrial septal communications can also be the primary lesion on rare occasions. This polyadenylation variant has been seen in three individuals with no abnormal heart phenotype in the population (n = 2504, phase 3 of 1000 genomes project; SNP rs545752658). Because of its presence in non-affected individuals, at present it is difficult to support a substantial causal role for this variant. All four variants were absent in the ethnically matched control sample cohort (n = 384, UK blood donors, Sigma-Aldrich) and no additional coding variations of *TPM1* were observed in the control cohort.

### 3.2 Investigating the effect of a novel splice-donor site mutation in vitro

To assess the functional consequence of a novel *TPM1* splice site mutation, two constructs were created, spanning the 5′ upstream region of *TPM1* to exon 2a, using patient A and a control's genomic DNA (Fig. 1E). Constructs contained either the IVS1 + 2T > C splice site mutation, or wild-type *TPM1*. RT-PCR showed that the wild-type produced a transcribed *TPM1* product of 307 bp whilst no concurrent product was seen for the splice-site mutation construct (n = 3; Fig. 1F).

### 3.3 In silico analysis of missense mutations I130V and S229F in TPM1

The I130V mutation results when an adenine in exon 4, which is highly conserved, is replaced with guanine. Both isoleucine and valine are non-polar at physiological pH. However, the amino acid substitution is located in the *d* position of the seven residue heptad repeats. Additionally, the mutation is located within the fourth α-zone of TPM1.

**Fig. 2:**
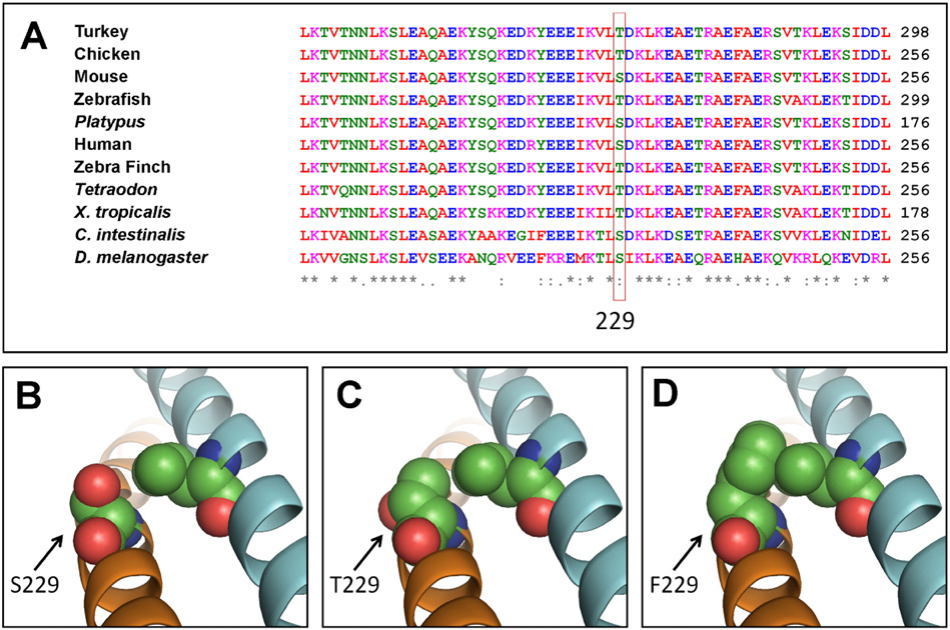
Multi-species amino acid alignment of a TPM1 segment and molecular modelling of TPM1 demonstrating alterations at residue 229. A. Multi-species alignment of orthologues of TPM1 showing the conservation of residue 229 (red rectangle). In this position, serine or threonine are present in mammals, fish, amphibians, insects and ascidians. B–D. Homology molecular models showing the structure of the residues serine (B), threonine (C) and phenylalanine (D) in position 229 of TPM1. Serine or threonine, which are conserved, allow proper interaction between two TPM1 molecules forming coiled-coil dimers whereas, phenylalanine with its bulkier side chain, encoded by the S229F allele, prevents that interaction, by steric clash between the two molecules.

The S229F mutation occupies a conserved position through evolution, as shown in Fig. 2A, where serine or threonine exist in birds, fish, mammals, *X. tropicalis*, *Drosophila* and *C. intestinalis*. Both residues have small, polar side chains. S229F mutant allele encodes a protein in which a phenylalanine residue occupies this position. A molecular homology model of the S229F TPM1 predicts that the much bulkier, non-polar side chain of phenylalanine would impair binding of two TPM1 peptide molecules that form the coiled-coil tropomyosin filament by steric hindrance (Fig. 2B–D).

### 3.4 Analysis of I130V and S229F missense mutations in the embryonic chick heart

**Fig. 3:**
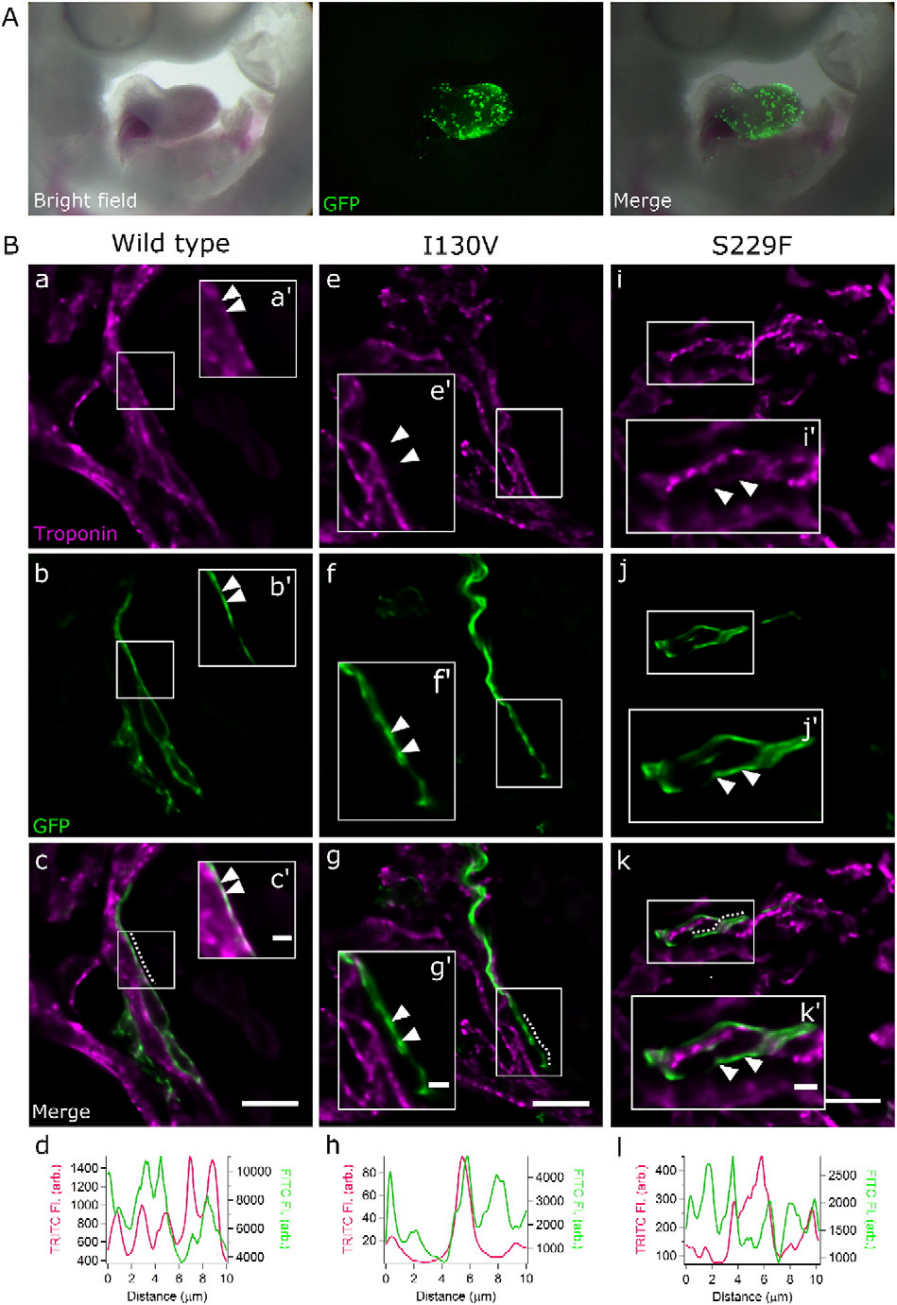
Whole embryonic hearts transfected with GFP-tagged constructs and visualized on a DeltaVision microscope. A. Embryo microinjected with GFP-tagged constructs and harvested 48 h post-injection (n = 4 per group). GFP fluorescence was restricted to the heart. B. Photomicrographs of sarcomeres transfected with control (a–c), GFP/TPM1-I130V (e–g) and GFP/TPM1-S229F (i–k) constructs. The sarcomeres showing GFP expression (green; panels b, f, j) can be compared to the same region (arrows landmark the same positions) counter stained with the TRITC labelled troponin sarcomeric marker (magenta; panels a, e, i); merged images presented in c, g and k (magenta and green merge shows white). Boxed areas denote higher magnifications (a′–k′). Wild-type show coincidence of the GFP and the troponin marker (arrows in a′, b′ and c′; white shows overlap of similar green and magenta signals). This is supported by the fluorescence intensity plot (d) (obtained from region denoted with a line in c), with peaks for GFP and TRITC correlating. In contrast, with the I130V mutant, coincidence of the GFP construct was not observed with the troponin sarcomeric marker (e′, f′ and g′; i.e. the sarcomere is not evident at the arrows in e′ but is at the same location (arrows) in f′); in the intensity plot (h) correlation of TRITC did not occur with GFP (region denoted with dotted line in g). This was also true for the S229F mutant, where coincidence of GFP was not seen with troponin (i′, j′ and k′; supported by the intensity plot in l with region denoted with dotted line in k). Note that the low intensity peaks (see the scale for TRITC fluorescence in h with that in d) observed in the troponin channel in h come from fluorescence from adjacent z-planes, i.e. out-of-focus objects not apparent in the image. Similarly, the TRITC peak in l comes from a ‘crossing’ sarcomere. Images shown are representative of sarcomeric phenotypes for each experimental group. Scale bar in c, g and k is 10 μm, and in c′, g′ and k′ is 2 μm.

To investigate the functional consequences of the missense mutations, chick HH11 hearts were transfected and subsequently harvested at HH22; the GFP-tagged constructs were found to transfect successfully (Fig. 3A). Hearts transfected with either GFP/TPM1-WT, GFP/TPM1-I130V or GFP/TPM1-S229F constructs (n = 4 per group) were analysed with immunofluorescence for the sarcomeric structural protein, and *Z*-stack imaging of sectioned hearts was performed. Examination of single z-planes showed that cardiomyocytes successfully transfected; GFP/TPM1-WT had coincident localisation of the sarcomeric marker troponin T and GFP (n = 15 sarcomeres; the arrow landmarks in Fig. 3Ba, a′ compared to Bb, b′ and merged image Bc, c′; regions of peak troponin T and GFP signal show white, i.e. magenta plus green). The coincident punctate expression was further demonstrated by plotting the fluorescence intensity along sarcomeric sections (Fig. 3Bd).

Transfecting cardiomyocytes with GFP/TPM1-I130V led to GFP expressed in a punctate pattern but the troponin marker did not coincide (n = 13; see the positions marked with arrows in Fig. 3Be, e′ showing the absence of troponin T marker to those in Bf, f′ showing punctate GFP at the same location, and in the merged image Bg, g′). This was again confirmed with fluorescence intensity plots (Fig. 3Bh); note that the (low intensity) peaks observed in the troponin channel (Fig. 3Bh) originate from z-planes above or below the one shown, i.e. out-of-focus objects that are not apparent in the single-z-plane images. In addition, one sarcomere observed expressed the troponin marker in an alternating fashion to the GFP expressing TPM1-I130V construct. GFP/TPM1-S229F transfected cardiomyocytes appeared similar to the GFP/TPM1-I130V expressing sarcomeres, where troponin was not expressed in the same region for the majority of the sarcomeres analysed (n = 16; arrowed locations Fig 3Bi, j and k) and confirmed by fluorescence intensity plot (Fig. 3Bl). Further, as seen for TPM1-I130V, one GFP/TPM1-S229F sarcomere showed alternating expression of GFP and troponin (n = 1; Supplementary Fig. 1).

### 3.5 Looping defects seen upon TPM1 morpholino treatment

To assess TPM1 requirement during heart development, embryos were morpholino-treated at HH10/11 and harvested at HH19. Survival rates for the untreated (UT; n = 81), standard control (SC; n = 92) and TPM1-morpholino treated groups (TPM1-MO; n = 123) were 91.4%, 89.1% and 88.6%, respectively (*P* = 0.98). Following ‘morpholino uptake’ determination in surviving embryos, 68.3% SC and 88.1% TPM1-MO treated embryos were positive (*P* = 0.76).

**Fig. 4:**
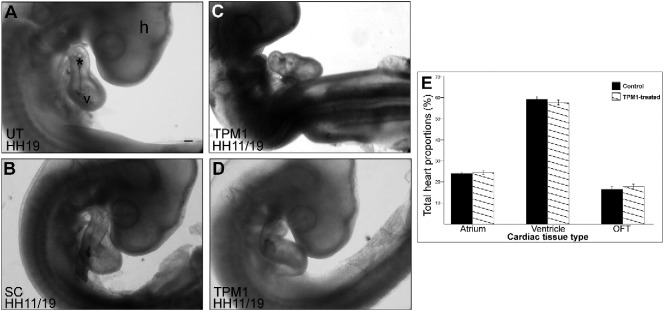
TPM1-morpholino treatment leads to abnormal looping. Embryos were treated at HH11 and harvested at HH19 (HH11/19) for both the control (A, B) and TPM1-MO (C–E) groups. External phenotypic analysis shows a subset of TPM1-MO embryos displayed looping defects (n = 4; D, E) in comparison to control and normally looped TPM1-MO embryo hearts (n = 137 and 96, respectively; A–C). Stereological analysis revealed normal cardiac tissue proportions (atrium, ventricle and outflow tract) between control and TPM1-MO embryos (n = 11 and 13, respectively; F). Scale bars: 500 μm. h, head; asterisk, outflow tract; OFT, outflow tract; v, ventricle.

Embryos were externally analysed upon harvesting. The UT and SC groups all appeared phenotypically normal, hence the groups were pooled (n = 137; Table 1; Fig. 4A, B). Following application of TPM1-MO, the external phenotype of embryonic hearts appeared normal for the majority of embryos (Fig. 4C; 92/96, 96%). In the 4% that appeared abnormal (4/96), the hearts were dextrally distorted, indicating a looping defect (Table 1; Fig. 4D, E). Stereological analysis showed no proportional differences of cardiac atrium, ventricle or OFT between the control and TPM1-MO groups (*P* = 0.3; Fig. 4F).

**Table 1:** Summary of phenotypic analysis following treatment with TPM1 morpholinos.

Stage^a^	Embryo type^b^	Heart shape			Atrial septum			Trabeculation			Total^c^ int
		Ab looping	Normal	Total^c^ ext	Double	Reduced	Normal	Absent	Reduced	Normal	
HH11/19	Control	–	137	137	–	–	60	–	–	60	60
	TPM1 ATG	2	34	36	1	11	17	1	12	16	29
	TPM1 E1I1	1	28	29	–	9	17	–	5	21	26
	TPM1 E4I4	1	30	31	–	7	7	–	8	6	14

Assessment of heart shape based on qualitative analysis of gross morphological features.

Qualitative assessment of septation and trabeculation obtained from serial histological sections.

Ab, abnormal; ext, externally; int, internally.

a Stage of development when morpholino treatment/harvesting performed.

b Control embryos include standard control and untreated; TPM1 denotes embryos treated with tropomyosin 1 ATG or splice site morpholino.

c Total number of embryos analysed.

### 3.6 TPM1 is required for normal atrial septa and trabeculae formation

**Fig. 5:**
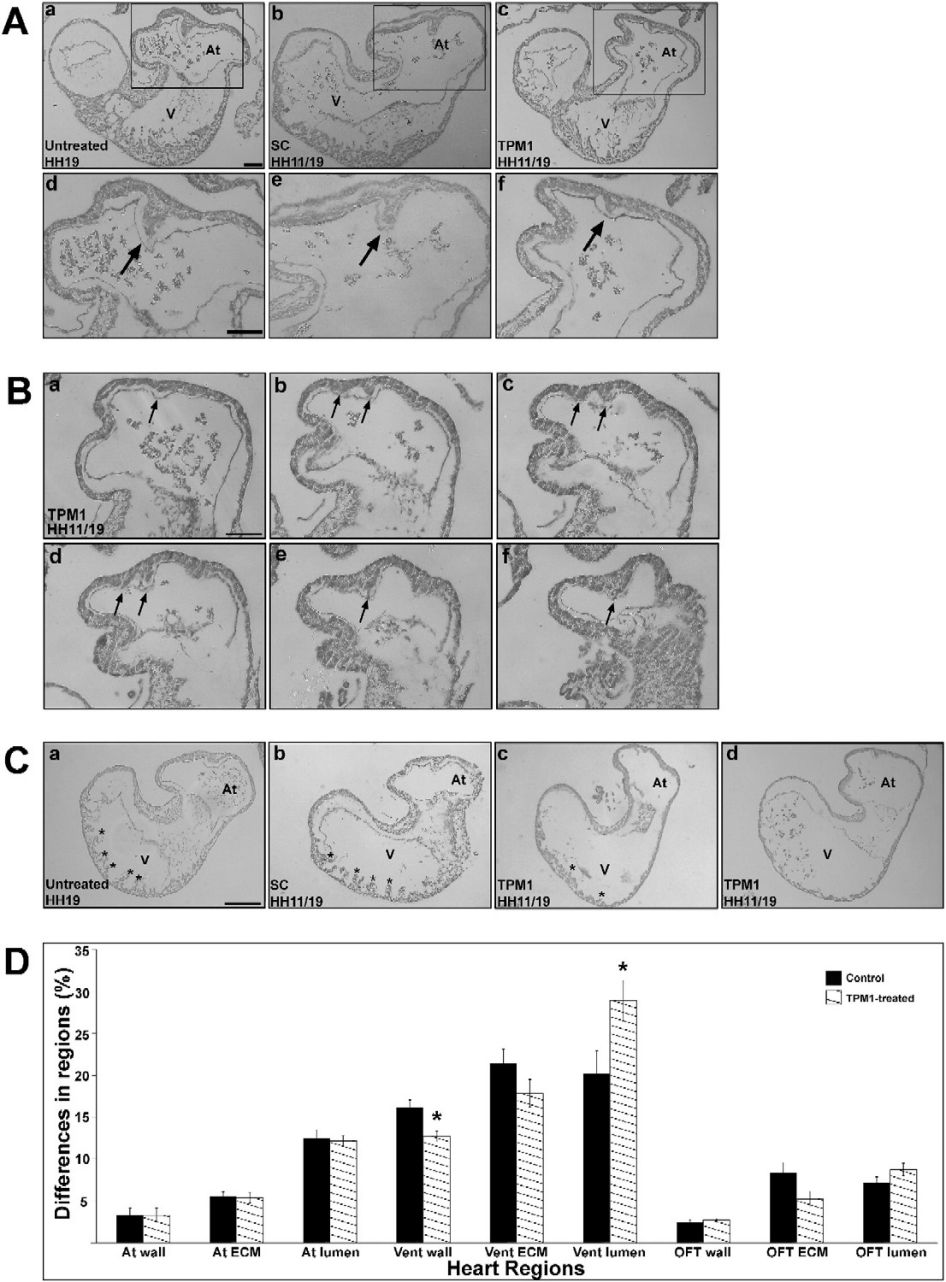
TPM1 morpholino treatment results in abnormal atrial septa and trabeculae formation. A. Untreated (UT), standard control (SC) (a, b, d, e), and TPM1-MO (c, f) hearts were imaged at low (top panel) and high (lower panel) magnifications. The septum in the TPM1-MO heart (c; n = 27/69) was smaller compared to control hearts (a, b; n = 60). Scale bars a–c: 100 μm; d–f: 200 μm. B. Serial sections through one TPM1-MO embryo revealed a double septum. One septum originated from the atrial wall (a), followed by a second, smaller septum (b, c, d). Deeper sections showed the fusion of these two septa (e, f). Scale bar: 200 μm. Arrows denote septa. C. UT and SC embryos contained normal trabeculae projecting into the ventricular chamber (a, b; n = 60). In contrast, TPM1-MO hearts showed reduced trabecular size and number (c; n = 25/69) or complete absence of trabeculae (d; n = 1/69). Asterisks denote trabeculae. Scale bar: 100 μm. D. Stereological analysis of heart regions revealed that TPM1-MO hearts had decreased ventricular wall and trabeculae proportions (denoted as Vent wall) and an increased ventricular lumen size compared with controls. Mean ± SEM; *P < 0.05. Asterisk, trabeculae; At, atrium; ECM, extracellular matrix, OFT, outflow tract; V, ventricle.

Embryos were transversely, serially sectioned to analyse internal structures. The septum appeared to develop normally (extending from the atrial wall roof towards the AV canal) in all controls (n = 60; Table 1; Fig. 5Aa, b, d, e). In 41% of the morpholino-treated embryos, the septum was small and resembled a knuckle shaped outgrowth from the atrial myocardium (n = 28/69; Table 1; Fig. 5Ac, f). One heart displayed a remarkable septal phenotype with the initiation of a second atrial septum from the dorso-cranial wall (Table 1; Fig. 5B). Sequential tissue analysis revealed the appearance of the septum proper (Fig. 5Ba), adjacent to a second septum (Fig. 5Bb). These septa progressed in size, with the septum proper achieving close to normal septal length (Fig. 5Bc, d). Finally, when approaching the posterior atrial wall, the septa fused to form one common septum (Fig. 5Be, f). Stereological data showed that the atrial septum represented 0.61 ± 0.13% in the control hearts (n = 11) and 0.42 ± 0.11% in the TPM1-MO hearts (n = 13), a 30.18% decrease in atrial septation compared to control groups.

Trabeculae appeared normal in control groups (n = 60; Table 1; Fig. 5Ca, b). In TPM1-MO hearts, 38% had abnormal trabecular development, with a severe reduction in number and size (n = 26/69; Table 1; Fig. 5Cc). One heart completely lacked defined trabeculae (Fig. 5Cd). Stereological analysis showed the ventricular wall and trabeculae in control hearts represented 16.18 ± 0.87% of the hearts proportion, while the TPM1-MO ventricular wall and trabeculae accounted for 12.68 ± 0.60%; a decrease of 21.64% (*P* = 0.003; Fig. 5D). Consistent with this, the ventricular lumen for the control and TPM1-MO hearts accounted for 20.17 ± 2.72% and 28.90 ± 2.37% respectively, an increase of 43.25% in TPM1-MO groups (*P* = 0.024; Fig. 5D). Embryos that presented with the abnormal looping phenotype all presented with abnormal atrial septa or reduced trabeculae.

From all the embryos assessed in this study, 55% (38/69) had either an abnormal septum or reduced trabeculae, while 23% (16/69) presented with both of the abnormal phenotypes.

### 3.7 TPM1 morpholino treatment results in cell death

**Fig. 6:**
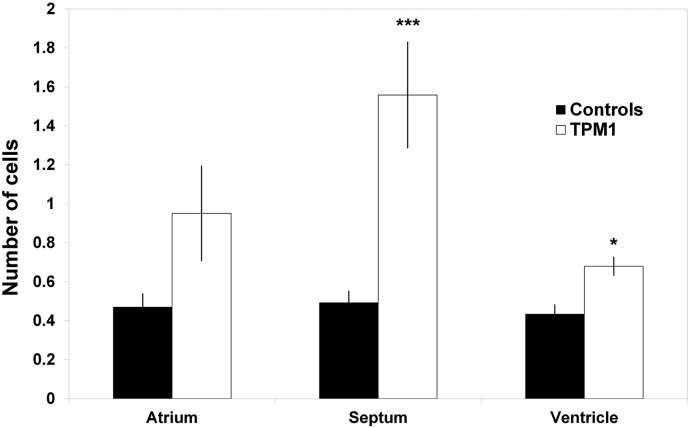
Apoptosis increased in the atrial septum and ventricular wall of TPM1-MO hearts. Apoptosis was measured in UT, SC (pooled as controls; n = 6) and TPM1-MO hearts (n = 7). Apoptosis was significantly increased in the atrial septum and ventricles of TPM1-MO hearts, *P < 0.05 ***P < 0.001.

Apoptosis was quantified in TPM1-MO hearts from a total of 28,103 cells. No differences in apoptosis were observed between UT and SC hearts, and were pooled into one control group (n = 3 for both UT and SC; *P* > 0.087). In the atrial wall, 0.47 ± 0.07% of the control and 0.95 ± 0.24% of the TPM1-MO heart cells were apoptotic (n = 7 TPM1-MO hearts; *P* > 0.05; Fig. 6). In the atrial septum, apoptosis accounted for 0.49 ± 0.06% cells in control and 1.56 ± 0.27% in TPM1-MO treated hearts (*P* < 0.001; Fig. 6). Finally in the ventricular wall, 0.43 ± 0.05% of the cells were apoptotic in control hearts compared to 0.68 ± 0.05% in TPM1-MO hearts (*P* = 0.013; Fig. 6).

Cell proliferation was analysed in the same hearts from a total of 47,747 cells. No significant differences in PCNA positive cells were seen between the SC and UT hearts (*P* > 0.085) and were pooled. Cell proliferation appeared normal in the atrial wall, atrial septum and ventricular wall in the TPM1-MO hearts when compared with controls (*P* > 0.271).

### 3.8 TPM1 morpholino treatment reduces sarcomeric maturity

**Fig. 7:**
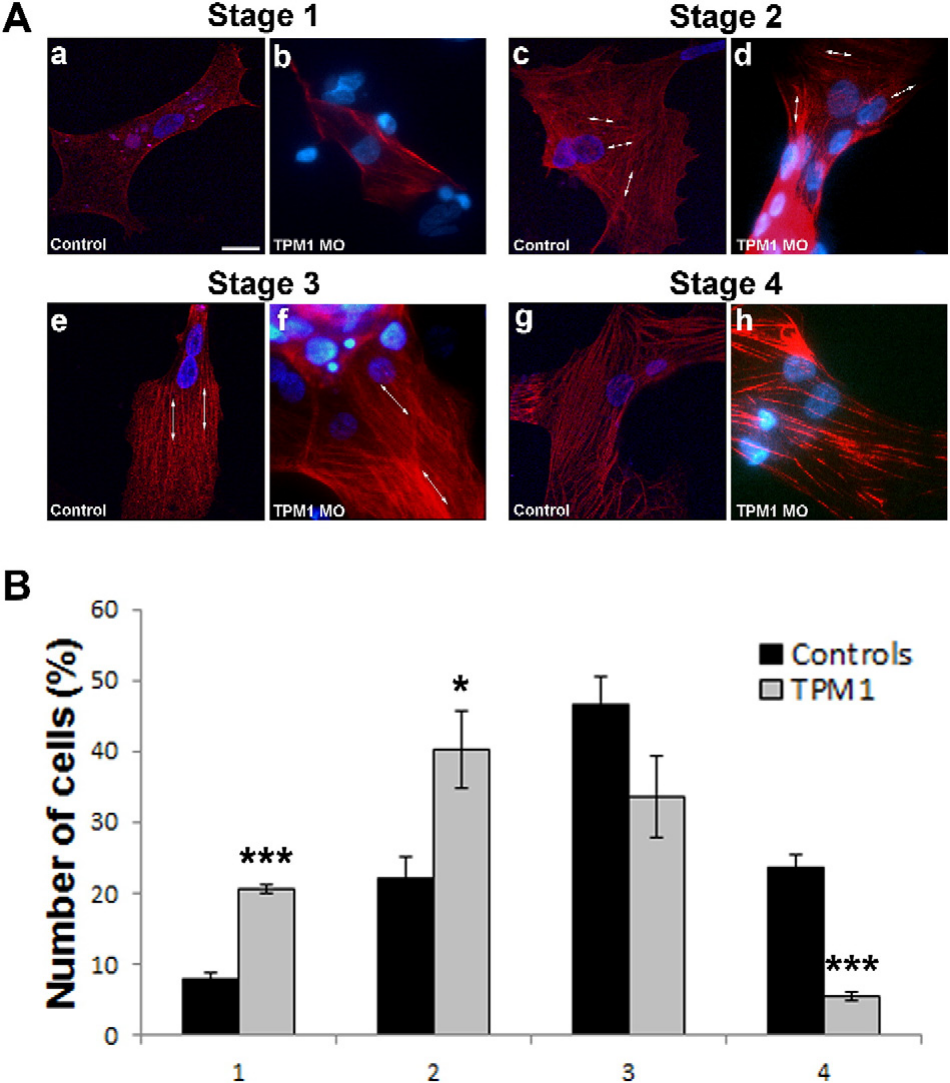
TPM1-MO treatment leads to immature sarcomere assembly. A. Sarcomere assembly was categorized into 4 stages: stage 1 immature myofibril assembling at the periphery of the cell with no fibril structures present (Aa and b); stage 2 fibres present in a disorganised fashion (Ac and d); stage 3 thin but organised fibrils (Ae and f); stage 4 fully developed thick fibrils running across the cell (Ag and h). Control indicates cells that were not treated with TPM1 morpholino, while TPM1-MO indicates cells treated with morpholino for 48 h. Scale bar: 16 μm. B. TPM1-MO treatment resulted in significant increases in stage 1 and stage 2 immature cells, significant decreases in the number of stage 4 cells and reduced (but not significantly) number of stage 3 cells. *P < 0.05 ***P < 0.001.

The effect of TPM1-MO on sarcomere assembly was investigated using cardiac cell micromass. Control and TPM1 treated cardiomyocytes were classified into four stages depending on the degree of sarcomeric maturity (see Section 2.14; Fig. 7A). Stage one consisted of cells with positive staining around the periphery of the cell with identifiable sarcomeric structures not seen (Fig. 7Aa, b). Stage two cardiomyocytes had thin and disorganised sarcomeres (Fig. 7Ac, d). Stage three cardiomyocytes had thin, organised sarcomeres that run parallel to one-another (Fig. 7Ae, f). Finally, stage four cardiomyocytes contain mature, thick sarcomeres (Fig. 7Ag, h).

UT and SC cells showed no difference in any of the classifications and were pooled into one control group (n = 753 cells; *P* > 0.217). When the control group was compared with the TPM1-MO group (n = 273), the TPM1-MO cells had significantly more sarcomeres at stage 1 and 2 (*P* < 0.001 and *P* = 0.012, respectively; Fig. 7B). No difference was seen at stage 3 between the groups (*P* = 0.120), however, the number of TPM1-MO cells with stage 4 sarcomere assembly was significantly reduced (*P* < 0.001).

Following tissue integrity investigations, no differences between TPM1-MO (Supplementary Fig. S2; n = 8 TPM1) and control (Fig. S2A; n = 3) hearts were observed when using sarcomeric (alpha-actinin; red label in top panels Fig. S2A&B) or plasma membrane (beta-catenin; green label in top panels Fig. S2A&B) markers. At the ultrastructural level, the controls and TPM1-MO appeared indistinguishable from each other when cellular structures such as myofibril assembly, presence of desmosomes and mitochondria, were analysed (n = 9; Fig. S2C).

### 3.9 TPM1-MO hearts displayed altered action potentials

**Fig. 8:**
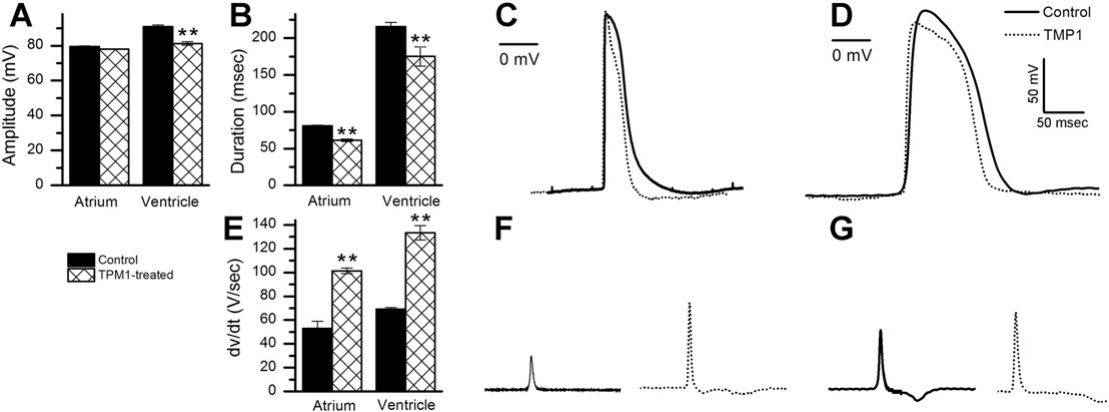
TPM1-MO treatment alters action potentials in cardiomyocytes. A–D. Graphs showing changes in AP amplitude (A) and duration (B) between atrial and ventricular cardiomyocytes in control and TPM1-MO hearts. Representative AP traces from atrial (C) and ventricular cardiomyocytes (D) for control (black traces) and TMP1-treated (dotted traces) hearts were superimposed to show amplitude and duration changes. E–G. Graphs showing changes in the rate of depolarisation (δv/δt) of the atrial and ventricular cardiomyocytes from control and TPM1-MO hearts (E). Representative AP traces of the maximal δv/δt from both atrial (F) and ventricular (G) cardiomyocytes for control (black traces) and TPM1-MO hearts (dotted traces) are shown.

Intracellular recordings were obtained from single spontaneous beating cardiomyocytes from the atria and ventricles of TPM1-MO (n = 549 and 398, respectively) and control (n = 187 atria and 177 ventricular) hearts. APs from TPM1-MO ventricular cardiomyocytes had significantly decreased amplitudes, with atrial cell amplitudes comparable to controls (Fig. 8A; representative traces for atrial cells Fig. 8C and ventricular cells Fig. 8D). In contrast, both atrial and ventricular cells from TPM1-MO hearts exhibited shorter AP durations (Fig. 8B; representative traces in Fig. 8C&D respectively) and increased maximal δv/δt compared to controls (Fig. 8E; representative traces Fig. 8F&G). APs were altered in 100% of TPM1-MO cells analysed.

## 4 Discussion

TPM1 is a parallel double-stranded α-helical coiled-coil protein, which binds to the long grooves of actin, making it crucial in actin regulation and stability [31]. It also functions with the troponin complex in response to Ca^2 +^ [17]. Two isoforms of the *TPM1* gene, *TPM1α* and *TPM1κ*, are expressed in equivalent amounts in both the fetal and adult human heart and differ by the inclusion of either exon 2b or exon 2a, respectively. At the protein level, however, TPM1α accounts for about 92% of total TPM protein, with TPM1κ and TPM2α (protein product from the *TPM2* gene) making up the remainder [32], [33]. TPM1 has recently been associated with CHDs in a two year old female with Ebstein anomaly, left ventricular non-compaction, pulmonary hypertension and end-stage heart failure [14]. The de novo mutation was found in a highly conserved region of exon 4, D159N, and an actin binding region of TPM1. However, this mutation was not associated with isolated CHD's, unlike the mutations reported in the current study.

To determine a role for *TPM1* in human cardiogenesis, 380 individuals with isolated CHD's were screened. Given that we and others have shown that Mendelian CHD often shows variable expressivity [15], [34] including malformations without obvious causal or taxonomic relation even in the presence of an identical mutation, we decided to include patients with any type of CHD. Four novel mutations were detected; two missense, a splice site and a polyadenylation signal variant. T > C substitutions in the splicing donor-site are known to cause human disease when present in other genes [35], [36]. The most likely outcome of these mutations is intron1/2 inclusion in mature mRNA, the use of cryptic donor sites in the same intron or in the adjacent exon, or exon skipping [37], [38]. In this study the splice site mutation IVS1 + 2T > C, found in patient A with TOF, was analysed in vitro; it did not result in the use of cryptic donor sites (first one located 137 bases downstream of the donor-splice site), since no RT-PCR product was produced (Fig. 1F). Therefore, the donor-splice site in the first intron of *TPM1* is essential for the production of normal RNA products.

The I130V mutation is located within the *d* position of the heptad repeat of TPM1, the region responsible for giving TPMs their coiled-coil property [39]. In addition, the I130V mutation is located within the fourth α-zone of the TPM1 protein, which has been proposed to interact with subdomains 1 and 3 of F-actin, in the context of the sarcomere [31]. Therefore, it is postulated that interruptions in these binding regions may affect the stability of the coiled-coil structure of TPM1 [40]. However, the side-chain of the valine residue encoded by the derived allele is smaller than that of the isoleucine encoded by the ancestral allele, and both are predicted to adopt the same orientation, *i*. *e*. towards the opposite TPM1 molecule of the dimer, so they would form part of the interface surface between them. Thus, it is unlikely that this mutation compromises the stability of the coiled-coil by steric hindrance. Nevertheless, the space left by a smaller valine side-chain could alter dynamics of the coiled-coil dimer through the contractile cycle, but, in order to support this notion further, a dynamic structural model of the TPM1 dimer would be required, which is not currently available. The S229F mutation occupies a conserved position through evolution, where either the small, polar residues serine or threonine exist (Fig. 6A). The S229F mutant allele encodes a protein in which a phenylalanine residue occupies this position. As shown in Fig. 2B–D, the S229F substitution is predicted to impair binding of TPM1 peptides since phenylalanine is non polar and much bulkier than a serine residue. Both of these novel missense mutations, therefore, may affect the stability of the TPM1 structure and stability within the sarcomere. As mentioned in Section 3.1, we were unable to assess the de novo status of the I130V, S229F and splicing site variants, as we lack critical parental samples. Nevertheless, we [5], [15] and others [34], [41] have shown that mutations causing Mendelian CHD often show incomplete penetrance and variable expressivity which means that, even in the case of a variant present in an unaffected parent, this could be pathogenic.

*In ovo* transfection using TPM1 constructs containing the novel missense mutations, I130V and S229F, revealed the absence of co-localisation between the two thin filament proteins, troponin T and the GFP/TPM1 mutant construct (Fig. 3Be–g, i–k). However, in the GFP/TPM1-WT sarcomeres, both proteins were always co-localised (Fig. 3Ba–c); therefore, the phenotypes seen were specific to the mutant TPM1 protein that was being expressed in the chick heart. Previous studies investigating the central region of TPM1 (residues 145–235) have shown its interaction with the troponin complex is intrinsically unstable [42], [43]. Alterations within the TPM1 central region are believed to affect Ca^2 +^-mediated activation of the thin filament, because of its interaction with the troponin complex. This in turn would affect the terminal ends of TPM1 (where TPM1 binds to the next TPM1 molecule) and affect its contractile function [44], [45]. Substitution of a negatively charged glutamic acid at position 229 of TPM1 (S229E) increased the distance between the helical interwinding of TPM1 molecules and affected cardiac contractility [46]. Therefore, subtle changes in TPM1 conformation can have dramatic effects within the sarcomere. We propose that these novel missense mutations result in alterations to the coiled-coil structure of TPM1 and may affect its incorporation into the thin filament and/or its association with other thin filament proteins [47].

The alternating expression of troponin T with GFP/TPM1-I130V and S229F observed in one sarcomere may be explained by the close association of two adjacent sarcomeres winding or twisting around each other. This is consistent with the absence of this alternating expression in wild-type sarcomeres, which showed only GFP and troponin marker co-expression.

The variant detected in the 3′ untranslated region in patient D with two ASDs and dilated right chambers modifies the first base of the sequence of the polyadenylation signal (AATAAA/GATAAA). Mutations of the polyadenylation signal can cause human disease [48], and specifically, has been shown that this particular substitution reduces the efficiency of the cleavage of the mRNA precursor at the polyadenylation site to approximately 30% of the wild-type sequence whereas the polyadenylation itself is reduced to 11% [49]. However, the effect of these mutations could be potentially ameliorated by the use of alternative downstream polyadenylation signals. Interestingly, the polyadenylation signal variant seen in our study is also located within an experimentally validated binding-site for microRNA-21 which downregulates TPM1 expression [50]. MircroRNA-21 plays a role in cardiac disease, and impairment of its function resulted in regression of cardiac hypertrophy and fibrosis in a mouse model of left ventricular overload [51]. Nevertheless, the variant occurs in the 3´UTR segment that binds the 3′ end of miR-21, and typically, mismatches in such regions do not modify strongly the affinity of microRNAs. Given that this variant has also been found in unaffected subjects, this suggests a weak correlation with the presence of cardiac malformation. Therefore, with the evidence available, the pathogenic potential of the variant detected in the 3′ untranslated region is uncertain.

From ours and previous studies, we can hypothesize that mutations in genes for proteins of the thick (i.e. MYH6) [15] and thin filaments (i.e. ACTC1 [7] and TMP1, the present report) of the sarcomere expressed in the human developing heart can cause cardiac malformation, supporting the notion that early intra-cardiac blood flow has evolved as an important epigenetic factor contributing to adequate cardiogenesis [52].

Many isoforms of *TPM1* exist through alternate splicing. Two striated isoforms are known to be expressed in the developing chick heart, *TPM1α* and *TPM1κ*, as well as smooth muscle and fibroblast isoforms (expression profiles were conducted on a 4-chambered and relatively mature heart at day 10 and 15 of embryonic development) [53]. This expression suggests roles for this actin-binding protein in the contractile system of both striated and smooth muscles cells, and in the cytoskeleton of non-muscle cells were it has a stabilising function. Targeted manipulation of *TPM1* was performed early in chick cardiogenesis, at HH10/11 or 2 days post-fertilisation, to gain insight to the role of *TPM1* during early heart development. Three different morpholinos were used to target different regions, and the resulting phenotypes remained consistent between these morpholinos. Morpholinos have come under criticism recently, due to off-target effects in zebrafish [54]. Thus we conclude that we did not observe off-target effects in this study. However, in order to study the role of TPM1 in specific lineages during development, cardiac-specific knockout of *TPM1* in these lineages will need to be performed in the mouse, with such specific markers not yet available.

Although a *Tpm1*^−/−^ mouse has been created, embryos died between E8.5–11.5 and were only recently characterized [55], [56]. These homozygous mice had thin ventricular walls with fewer, thinner trabeculae, enlarged intracellular spaces and extended cellular processes with smaller adherens junctions and myofibrils failed to form [56]. Since the *Tpm1*^−/−^ mouse dies early in development, and phenotypic analysis was conducted as the embryos were already undergoing resorption [56], it calls to question whether or not the observed phenotypes were down to the absence of *Tpm1*, or because the embryos were fated. Therefore, morpholinos can be a useful tool if carefully controlled, as they can be used later and at precise stages.

Although no individual phenotype had 100% penetrance, 58% of TPM1-MO embryos analysed internally showed abnormal phenotype(s). Of those analysed externally, 4% of embryos had an abnormal phenotype and also had reduced trabeculae and/or abnormal atrial septation. Stereological analysis revealed an underdeveloped ventricle, with reduced ventricular wall and trabeculae, and increased ventricular lumen size in TPM1-MO embryos. In addition, stereological analysis indicated that the atrial septum was also reduced in size, although this could not be statistically confirmed since the septum is small and did not reach the optimal number of grid points (63 out of a required 200 points were counted) [28]. These phenotypes may be explained by the statistically increased apoptosis in these regions (Fig. 6).

TPM1-MO treatment resulted in increased immature myofibrils. In the *Tpm1*^−/−^ mouse, myofibril assembly was ablated and thick and thin filaments failed to interact [56]. Similarly, the *cardiac* mutant axolotl heart had reduced *Tpm1*, resulting in failure of myofibrils to assemble [57], [58]. In this study, sarcomere assembly was initiated but myofibrils remained nascent; however morpholino treatment was performed after the initial heart tube had formed and therefore, TPM1 was already present in the heart. Conversely, in the *Tpm1*^−/−^ mouse and *cardiac* mutant axolotl, Tpm1 is absent or diminished from the offset therefore leading to a more severe phenotype.

Intracellular recordings from atrial and ventricular cells treated with TPM1-MO revealed altered APs. Interestingly, 100% of the recorded cells had affected APs, while only 58% of the TPM1-MO treated hearts displayed morphological abnormalities. This may indicate that the contractile machinery of the cells may be more sensitive to TPM1-MO treatment and the structural phenotype may be a secondary defect; the abnormal contraction leads to changes in haemodynamics and hence to developmental defects [59], [60]. The increased δv/δt indicates an increase in active Na^2 +^ channels during depolarisation of the AP, while the decrease in amplitude and duration suggests a decrease in L-type I_Ca_^2 +^ channels and an increased number of active I_K_^+^ channels [61]. In embryonic myosin heavy chain (eMHC) morpholino treated hearts, APs were also affected with decreased amplitudes and increased durations and δv/δt [27]. There was a dramatic change in Ca^2 +^ transients which prolonged the duration of the AP. Therefore, investigating the transients of ions within TPM1-MO treated hearts may elucidate the cause of the abnormal APs.

Previous studies reported that alterations in cardiac conductivity have a morphological effect on cardiogenesis. Connexins (gap junction proteins) ensure the propagation AP throughout cardiac muscle [62]. In the *Connexin40* deficient and null mouse, electrical conduction is remarkably impaired [63] and animals displayed VSD (opening between ventricular chambers), double-outlet right ventricle, TOF, endocardial cushion defects, bifid atrial appendage and aortic arch defects at an incidence of 18% and 33%, respectively [64]. Obvious changes in the contraction patterns of TPM1-MO hearts were not observed, although on-going experiments using atomic force microscopy may assist in evaluating such possibilities.

## 5 Conclusion

To date, a range of genes have been associated with isolated CHDs, from transcription factors, ligands and their receptors to structural proteins [65], [66], with mutations in these structural proteins also associated with cardiomyopathies [67], [68]. It is of note that some mutations in these genes have been associated with a wide spectrum of CHDs, which may occur as a single defect or in combination, such as ASDs, transposition of the great arteries, TOF and VSD [5], [6], [7], [15]. This study demonstrates that *TPM1* plays a crucial role in cardiogenesis, both structurally and physiologically. Functional analysis of novel human mutations has provided proof that *TPM1* is a candidate gene worthy of further screening for cardiovascular disorders.

## Funding

This work was supported by British Heart Foundation [grant numbers PG/04/022/16760 and PG/06/021/20345 to S.L., RG/07/010 and RG/13/10/30376 to J.D.B.] and American Heart Association [grant number AHASDG0530140N to L.P.P.] grants.

## Appendix A Supplementary data

Supplementary material Image 1
