# Supplementary material for: Tropomyosin 1: Multiple roles in the developing heart and in the formation of congenital heart defects
Source: J Mol Cell Cardiol. 2017 May;106:1–13. doi: 10.1016/j.yjmcc.2017.03.006 (PMC5441184; doi:10.1016/j.yjmcc.2017.03.006)
Supplement: Supplementary file 1 — Supplementary material [file mmc1.docx]

**Supplementary Information**

**Supplementary Materials and Methods**

**Construct design for splice-site mutation**

Genomic DNA was obtained from the patient with a heterozygous splicing-donor site mutation at exon1-intron1. DNA was also obtained from an ethnically matched control. Consent was obtained from all participants and work was approved by local ethics committees. Specific primers were designed to the 5’ upstream region and exon2a of *TPM1* (forward primer 5’ggggggcaggagaaaaaag3’; and reverse primer 5’TTGGCGGCGGCCTCTTC3’) and PCR products were obtained using Extensor Long Range PCR Enzyme (Thermo Fisher Scientific) under the following conditions: 95^o^C for 5 minutes, and then 95^o^C 30 sec, 59.7^o^C 45sec and 72^o^C for 3 minutes for 30 cycles. Sense and antisense primers contained additional *NheI* and *KpnI* linkers, respectively. After digestion with *NheI* and *KpnI*, amplified genomic fragments were sub-cloned into mammalian expression vector pcDNA3.1(+) (Invitrogen). Constructs were sequenced to identify wild-type and splice-donor site mutation (Source Bioscience).

**Analysis of splice-site mutation**

The wild-type and splice-donor site mutation containing pcDNA vectors were transfected into COS7 cells using Polyfect Transfection reagent (Qiagen). For each clone, 3 transfection experimental repeats were performed. As a positive control, COS7 cultures were transfected with ‘empty’ pcDNA vector. Cultures were incubated at 37^o^C for 48 h, harvested, washed in PBS and stored at -80^o^C. RNA was extracted from the COS7 cells using Tri-reagent (Sigma) according to manufacturer’s instructions. The RNA was DNase I treated (Qiagen). Reverse transcription reactions were performed using 0.5 μg of RNA and random primers with SuperScript III Reverse Transcriptase (Invitrogen). Primers were designed to the 5’upstream region and exon2a of *TPM1* (forward primer 5’gtattggctgtcttgaggaatg3’; reverse primer 5’gagaagttgctgcgggtgt3’) to indicate whether transcription of the genomic insert had occurred. Primers designed to *β-actin* were used as a control (forward primer 5’ACTCCATCATGAAGTGTGACG3’; reverse primer 5’CATACTCCTGCTTGCTGATCC3’). Products were resolved on 2% agarose gels against Hyperladder I (Bioline).

**Preparation of TPM1 constructs containing I130V and S229F missense mutations**

RNA was extracted from human heart myocardium using Tri-reagent according to manufacturer’s instructions (Sigma). The RNA was DNase I treated (Qiagen). Reverse transcription reactions were performed using 1 μg of RNA and random primers with SuperScript III Reverse Transcriptase (Invitrogen). Full length *TPM1α* (852 bp) was obtained using specific primers (forward primer 5’ATGGACGCCATCAAGAAG3’; and reverse primer 5’TATGGAAGTCATATCGTTGAG3’). The PCR product was obtained using Phusion high fidelity DNA polymerase (NEB) under the following conditions: 98^o^C for 2 min, followed by 98^o^C for 30 s, 53.5^o^C for 30 s, 72^o^C for 30 s for 33 cycles, with a final extension time of 4 min at 72^o^C. Forward and reverse primers also contained additional *EcoRI* and *BamHI* linkers, respectively. The *TPM1α* PCR product was cloned using the StrataClone PCR cloning set (Agilent Technologies). Selected colonies were cultured overnight in LB broth, minipreped and sequenced (Source Bioscience). The *TPM1α* construct was digested with *EcoRI* and *BamHI* for 2 h at 37^o^C. The digests were run in a 0.8% agarose gel, bands extracted and purified using a GeneElute gel extraction kit (Sigma). The *TPM1α* digest was ligated into pEGFP using T4 DNA ligase (NEB) overnight at 16^o^C. The ligation mixture was transformed into DH5α competent cells and grown overnight on LB-Kanamycin agar plates at 37^o^C. Individual colonies were picked and inoculated in 5 ml of LB broth and grown overnight at 37^o^C. Cultures were minipreped using the GeneElute Plasmid Miniprep Kit (Sigma). The GFP/TPM1 construct was sequenced (Source Bioscience).

Each PCR sample reaction contained 1X Vent buffer, 0.5 µg of pEGFP-TPM1 construct, 0.5 µM of each mutagenic primer, 200 µM dNTP mix, 1U Vent DNA Polymerase (NEB) and ddH_2_O to make a final volume of 50 µl. For a control reaction, the primers were excluded and replaced with ddH_2_O. The PCR reaction was heated to 95.9^o^C for 3 min followed by 20 cycles of 95^o^C 45s, 65^o^C 1 min, and 72^o^C for 5 min. The final elongation time was at 72^o^C for 15 min. Each PCR reaction was purified using the GeneElute PCR Clean-up Kit (Sigma) and treated with *DpnI* (NEB) at 37^o^C for 5 h to digest the parental dsDNA. The entire reaction was then transformed into 100 µl of DH5α cells and plated on LB-Kanamycin agar as described in the previous section and chosen colonies were grown overnight in LB broth at 37^o^C. Each culture was minipreped using a Plasmid Miniprep kit (Sigma) and were sequenced using the T3 primer to ensure the mutations had been introduced (Source Bioscience).

**Immunostaining of transfected chick hearts**

Cryosections collected for immunohistochemistry were rehydrated in PBS for 10 min at room temperature. The sections were blocked with 5% normal goat serum prior to primary antibody incubations. Primary antibody mouse anti-troponin T (CT3; 1:100; Developmental Studies Hybridoma Bank) was diluted in 1% BSA in PBS and incubated on the tissue overnight at 4^o^C. Sections were washed in PBS and incubated with AlexaFluor 546 secondary antibody 1:2000 (Life Technologies). Sections were mounted using GeneTex Mounting Media (GeneTex).

**Analysis of I130V and S229F missense mutations in the embryonic chick heart**

Z-stacks were collected (200 nm spacing) using a DeltaVision Elite microscope system (GE Healthcare Life Sciences) with a x60 and 1.42 numerical aperture objective (Olympus). Images were recorded on a CoolSnap HQ2 camera (Photometrics). The ‘standard’ filter wheel was used. EGFP fluorescence was excited at 475 nm (bandwidth 28 nm) and the emission was recorded at 523 nm (bandwidth 36 nm). TRITC (Troponin) fluorescence was excited at 542 nm (bandwidth 27 nm) and the emission recorded at 594 nm (bandwidth 45 nm). Images of GFP and TRITC fluorescence were acquired sequentially for each z-stack. Image stacks underwent iterative restoration in Huygens Professional (Scientific Volume Imaging). Exposures ranged from 0.1 s to 0.4 s; it was necessary to vary exposure to prevent saturation of any pixels for iterative restoration but to still maintain similar signal levels across these fluorescently-heterogeneous sections. While it is not ideal, the changes in exposure that were made would *reduce* the phenotypes observed. For example, in Fig. 3B the exposure for the TRITC channel (troponin T sarcomeric marker) was approximately 3-fold higher for the I130V TPM1 mutant compared to the WT. This would therefore *reduce* the magnitude of the *observed* phenotype and not artificially enhance it. Subsequent analysis of images was performed in Fiji ([1](#_ENREF_1)). Representative field of view (see Fig. 3B) were selected by choosing a single z-plane that showed clear periodic Troponin sarcomeric marker. The contrast in these areas (for both channels) was then stretched to enhance the signals in the zoomed in regions (insets in Fig. 3B), this was necessary due to the large dynamic range of intensities in the restored images. Line profiles (of the iteratively restored, but non-contrast-stretched data) for the regions shown in Fig. 3B were generated in Fiji and prepared in Igor Pro (Wavemetrics, Oregon, USA). The final figure was prepared in Inkscape (http://www.inkscape.org/).

**Application of TPM1 morpholino**

Morpholino application was performed on fertile chicken eggs (*Gallus gallus*, Dekalb White strain; Henry Stewart) at Hamburger and Hamilton 10/11 ([2](#_ENREF_2)). When determining the optimal concentration of TPM1 morpholino, 125 µM, 250 µM or 500 µM were achieved by resuspending in equal amounts of 30% F127 pluronic gel (BASF) and HBSS. The phenotype was found to be absent at 125 µM, mild at 250 µM and present at 500 µM. Non-specific effects were not observed. As the phenotype showed greater penetrance at 500 µM, this was used for all future studies. 7 μl of 30% F127 pluronic gel (BASF) and morpholino mixture was pipetted directly onto the exposed embryo. During this procedure, all pipette tips, pluronic gel and morpholino was stored on ice to prevent thermogelation of the pluronic gel during application. The eggs were resealed with masking tape and reincubated without rotation at 38^o^C until they developed to HH19. This procedure was conducted for all TPM1 and SC morpholino treated embryos. Untreated controls were also included and underwent the same experimental procedure, but without application of the pluronic gel and morpholino mixture. All animal work was carried out according to national (UK Home Office) and institutional regulations and ethical policies.

**Phenotypic analysis**

Embryos were isolated at HH19 and fixed in 4% PFA in PBS. Those not used for immunofluorescence and electron microscopy (see below) were washed in 1xPBS twice followed by distilled water, dehydrated in an ethanol series and wax embedded in a transverse orientation. Serial 8 µm sections were taken (DSC1 microtome, Leica), dewaxed, rehydrated and stained with Mayers haemalum (Raymond Lamb). Unless otherwise stated, morphological analysis was performed double blind using an Axioskop 2 microscope (Zeiss).

**Immunofluorescence and electron microscopy**

For immunofluorescence, the hearts from PFA-fixed chick embryos were immersed in 1 mg/ml hyaluronidase (Sigma, UK) in PBS for 1 h at room temperature, permeabilised in 0.2% Triton X-100/PBS (PBT) for 45 min, blocked with 5% preimmune goat serum in 1%BSA/TBS for 30 min at RT, with primary antibody incubations overnight at 4°C. After six 20 min washes in 0.002% PBT, secondary antibody incubations were either overnight at 4°C or for 6 h at room temperature, further washed in PBT, and mounted for confocal microscopy. Analysis was carried out on a Zeiss (Germany) 510 confocal microscope with 405 diode, argon and helium neon lasers using a 63X/NA1.4 oil immersion objective. Data were processed in Image J (NIH). Antibodies: mouse anti-sarcomeric alpha-actinin, rabbit anti beta-catenin (Sigma); rabbit anti EH-myomesin ([3](#_ENREF_3)) and rabbit anti-MyBP-C ([4](#_ENREF_4)), Cy3-conjugated goat anti mouse immunoglobulins and Cy5-conjugated goat anti rabbit immunoglobulins (Jackson Immunochemicals). Nuclear staining was observed using DAPI, observed in the UV bandwidth (Sigma). Combinations of Cy2/Cy5 were used for lissamine and Cy3/Cy5 for fluorescein-tagged morpholinos. Hearts undergoing transmission electron microscopy were fixed in 3% glutaraldehyde in 1% cacodylate buffer, and post fixed in 1% osmium tetroxide for 1 h. The hearts were dehydrated in a graded series of ethanol and infiltrated with resin, which was polymerised for 48 h at 60^o^C. Samples were sectioned at 90 nm using a diamond knife and stained on copper grids using 50% methanolic uranyl acetate and Reynolds lead citrate. Sections were visualised using a FEI Tecnai 12 Biotwin TEM (FEI) at magnifications 4000X-43000X.

### Isolation of cell micromass from embryonic chick hearts, morpholino treatment and analysis

Fertile eggs were incubated for 5 days, washed with trigene and IMS, and placed in a clean and sterilised class II Laminar flow hood. Embryos were removed and the hearts were isolated and placed in 1:1 horse serum/HBSS on ice. Hearts were placed in trypsin/EDTA at 37^o^C, 5% CO_2_ for 20 min and the tissue was homogenised in 8 ml of culture media (10% heat inactivated FBS, 2 mM L-glutamine and 50 unitsml^-1^ penicillin / 50 µgml^-1^ streptomycin was added to 500 ml DMEM and nutrient mixture F-12 HAM). The suspension was centrifuged for 5 min at 1500 rpm, and the cell pellet resuspended with 2 ml of warm culture media. The cell suspension was seeded in a 24 well plate containing glass coverslips at a cell density of 4×10^5^ cells/ml in 500 µl of warm culture medium. 2.5 µl of TPM1 morpholino (10 mM) and 3 µl of Endo-Porter (GeneTools) was added to 500 µl of pre-warmed cardiomyocytes culture medium. SC morpholino and no treatment were used as controls. The culture media was removed from the cardiomyocyte micromass and the morpholino mixture was added to each well. Cells were left for 48 h in a CO_2_ incubator.

The culture media was removed after 48 h, cells were fixed in 4% PFA and permeabilized with 0.2% Triton-X 100 in PBS. CT3 mouse monoclonal antibody (DSHB) was prepared at a 1:50 dilution in 1% bovine serum albumin (BSA) in PBS and incubated overnight at 4^o^C. Alexa Fluor 546 goat anti-mouse secondary antibody (Thermo Fisher Scientific) was also prepared in 1% BSA in PBS at 1:1500. Cells were mounted on glass slides and visualized using the DMIRE2 inverted microscope (Leica). Cells positive for CT3 antibody were considered cardiomyocytes and cardiomyocytes positive for morpholino uptake were included in the study. Over 1000 cells were analyzed blind in 4 independent studies.

**Electrical activity**

Intracellular recordings from TPM1 morpholino treated embryos (HH11/19) and control spontaneous beating hearts were performed. Micropipettes (60-80MΩ) were pulled using a P-97 micropipette puller (Sutter Instruments, USA) and filled with a 3M KCl solution and connected to a Bridge Amplifier BA-1S (npi-Tamm). The electrical signals were digitized, stored and processed using a Digidata 1440A under Axotape 10 Software control (Molecular Devices Corp) and displayed in parallel on an oscilloscope (Instek). Recorded action potentials were analyzed by the maximal rate of rise (δV/δtmax), duration APD-APD50 and APD, amplitude, maximal diastolic potential and resting membrane potential ([5](#_ENREF_5)).

**Supplementary Tables**

**Supplementary Table 1. A list of primers designed covering the *TPM1* gene to complete dHPLC analysis.**

| **Exon** | **Upstream primer** | **Downstream primer** |
| --- | --- | --- |
| **1** | ccttgggaaagtacatatctgg | ccttccttctttccagacg |
| **2** | ccgtgtgttgtgtgtgtctaac | acagggagggagagagagaaa |
| **3** | tgtccttctggttctgtgc | cttccttcattgctctccag |
| **4** | gaagctaccaccctcactttc | ctccggtttattccaccag |
| **5** | gcatttgggaagttcagctc | gtacattgctccctggcttc |
| **6** | gatgggatctgatctctaccc | gctgtttagtcactgctctgc |
| **7** | gtgtatcactgcatgccttacc | tagcagagggtgaagtgaagg |
| **7a** | ctcctttttctctcctccttcc | gcctcttttgagcagctctt |
| **8** | gccatgagtagattgagctg | cctctgtgagaaatggcagt |
| **9** | ctgccatttctcacagagg | gacacacaggaagaatgtgc |
| **10** | ctcctttgggtcaaagatgc | ccaatccacacctgattgtg |
| **10a** | cctcactcaccctccatttc | aagggtgaacacaagagtgc |
| **10b** | gtctgtgtttcaagtgctctc | accctcatatgtgtgaagcag |
| **10c** | caagtgaccagttgctgtcc | agagaaacaagcaggcaagg |

**SupplementaryTable 2. A list of primers used for the mutagenesis.** In order to introduce the mutations found in the patients into the full length cDNA, primers were designed to the region. Highlighted in grey is the nucleotide change.

| **Primer** | **Primer sequence (5’ to 3’)** |  |
| --- | --- | --- |
|  | **Forward** | **Reverse** |
| **TPM1-I130V** | GAGGCATGAAAGTCGTTGAGAGTCGAGC | GCTCGACTCAACGAGCTTTCATGCCTC |
| **TPM1-S229F** | GAGATCAAGGTCCTTTTCGACAAGCTGAAGGAG | CTCCTTCAGCTTGTCGAAAAGGACCTTGATCTC |

**Supplementary Figures**

**
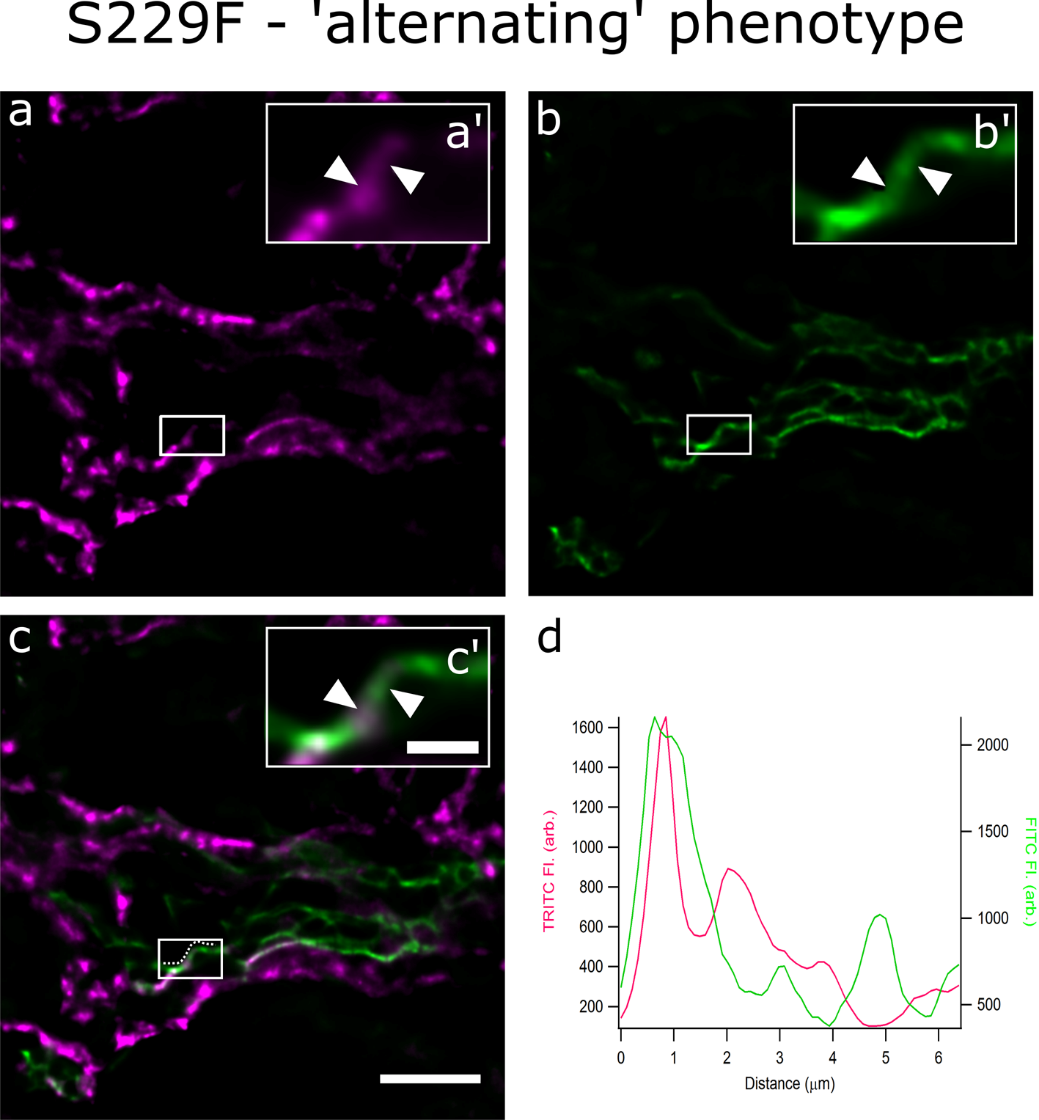
**

**Suppl­­ementary Figure 1. Whole TPM1-MO treated hearts transfected with GFP-tagged mutant construct** **and visualized on a DeltaVision microscope.**

Images of sarcomeres transfected with GFP/TPM1-S229F (a-c) construct. The sarcomeres stained with the TRITC labelled Troponin sarcomeric marker (a) can be compared to the regions expressing GFP (b); merged image in c. Boxed areas denote higher magnifications (a`-c`). In a defined area, the S229F mutant showed some correlation, but it appears to be alternating GFP and TRITC expression (a`, b` and c’); this was supported by the intensity plot (d). Images shown are representative of the ‘alternating phenotype’ seen in one area in each mutant. Scale bar in c is 10 μm (same for a,b) and in c` is 2 μm (same for a’,b’).


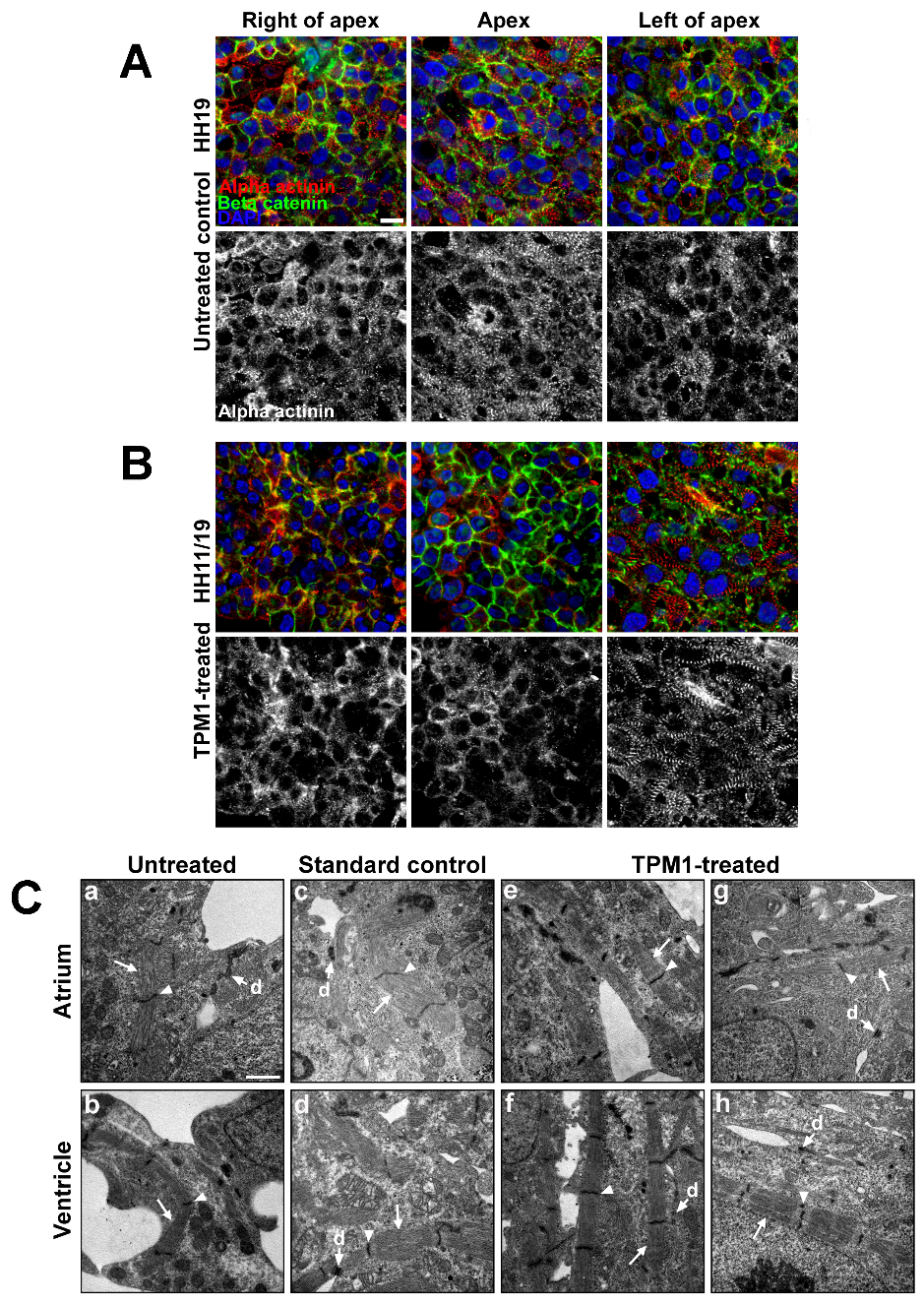


**Supplementary Figure 2. TPM1-MO treatment does not affect tissue integrity or sarcomere structure.**

**A and B**. Confocal photomicrographs of control (A) and TPM1-MO (B) hearts at HH19 were analysed (n=3 per group). Hearts labelled with beta-catenin (cell borders; green signal) and sarcomeric alpha-actinin (Z-discs; red signal and black and white images) with DAPI stained nuclei (blue signal). Three regions of each heart were imaged, the apex of the heart (middle column) and to the right and left of the apex. Control and TPM1-MO hearts showed no differences. Scale bar; 10 µm.

**C.** Electron microscopy was conducted on control (a-d; n=6) and TPM1-MO embryos (e-h; n=9) hearts. The atrial (a,c,e,g) and ventricular walls (b,d,f,h) were analysed separately. The intercalated discs (arrowheads), desmosomes (d) and muscle fibres (arrows) of the TPM1-MO groups were comparable to control groups. Scale bar: 2 µm.

**Supplementary References**

1 Schindelin, J., Arganda-Carreras, I., Frise, E., Kaynig, V., Longair, M., Pietzsch, T., Preibisch, S., Rueden, C., Saalfeld, S., Schmid, B. *et al.* (2012) Fiji: an open-source platform for biological-image analysis. *Nature methods*, **9**, 676-682.

2 Hamburger, V. and Hamilton, H.L. (1951) A Series of Normal Stages in the Development of the Chick Embryo. *J.Exp.Morphol.*, **88**, 49-49.

3 Agarkova, I., Auerbach, D., Ehler, E. and Perriard, J.C. (2000) A novel marker for vertebrate embryonic heart, the EH-myomesin isoform. *J Biol Chem*, **275**, 10256-10264.

4 Ahuja, P., Perriard, E., Perriard, J.C. and Ehler, E. (2004) Sequential myofibrillar breakdown accompanies mitotic division of mammalian cardiomyocytes. *J Cell Sci*, **117**, 3295-3306.

5 Polo-Parada, L., Zhang, X. and Modgi, A. (2009) Cardiac cushions modulate action potential phenotype during heart development [corrected]. *Dev Dyn*, **238**, 611-623.
